# Influence of the Maturity Stage on the Phytochemical Composition and the Antioxidant Activity of Four Andean Blackberry Cultivars (*Rubus glaucus* Benth) from Ecuador

**DOI:** 10.3390/plants9081027

**Published:** 2020-08-13

**Authors:** Iván Samaniego, Beatriz Brito, William Viera, Ana Cabrera, Wilma Llerena, Tissa Kannangara, Rubén Vilcacundo, Ignacio Angós, Wilman Carrillo

**Affiliations:** 1 Department of Nutrition and Quality, National Institute of Agricultural Research (INIAP), Panamericana Sur Km. 1, Mejía 170516, Ecuador; ivan.samaniego@iniap.gob.ec (I.S.); beatriz.brito@iniap.gob.ec (B.B.); william.viera@iniap.gob.ec (W.V.); 2 Facultad de Ciencia Químicas, Universidad Central del Ecuador (UCE), Av. Universitaria, Quito 170129, Av. Pichincha, Ecuador; accabrera@uce.edu.ec; 3 Facultad de Ciencias Pecuarias, Ingeniería en Alimentos, Universidad Técnica Estatal de Quevedo, Km 7 1/2 vía Quevedo-El Empalme, Los Ríos 120313, Ecuador; wllerenas@uteq.edu.ec; 4 Canadian Executive Service Organization (CESO), Toronto, ON M5G 1Z6, Canada; tkannang@shaw.ca; 5 Facultad de Ciencia e Ingeniería en Alimentos y Biotecnología, Universidad Técnica de Ambato (UTA), Av. Los Chasquis y Río Payamino, Ambato 180103, Ecuador; rd.vilcacundo@uta.edu.ec; 6 Departamento de Agronomía, Biotecnología y Alimentación, Universidad Pública de Navarra (UPNA), Edificio Los Olivos, Campus Arrosadia, 31006 Pamplona, Spain; ignacio.angos@unavarra.es

**Keywords:** *Rubus glaucus* Benth, blackberry, maturity stage, polyphenols, flavonoids, anthocyanins, antioxidant activity

## Abstract

Andean blackberries (*Rubus glaucus* Benth) are fruits rich in phytocomponents with high antioxidant activity. In this work, the changes in the total polyphenol content (TPC), the total flavonoid content (TFC), and the total anthocyanin content (TAC) of four blackberry varieties at three maturity stages (E1-25%, E2-50%, and E3-100%) were measured. The antioxidant activity (AA) was evaluated using the 2,2’azinobis-(3-ethylbenzthiazolin 6-sulphonic acid (ABTS) and ferric reducing antioxidant power (FRAP) methods. TPC and TFC content decreased with the increase in the maturity stage. The blackberry Brazos cultivar presented TPC values of 51.26, 38.16, and 31.59 mg of gallic acid equivalents (GAE)/g dry weight (DW) at E1, E2, and E3, respectively. The TAC and soluble solids increased with the increase in the maturity stage of the fruits. The Andimora variety at E3 presented a high TPC content, and the Colombiana variety presented a high TFC content. The blackberry Colombiana cultivar presented TAC values of 1.40, 2.95, and 12.26 mg cy-3-glu/100g DW at E1, E2, and E3, respectively. The blackberry Colombiana cultivar presented a high AA value at 1278.63 µmol TE/g DW according to the ABTS method and 1284.55 µmol TE/g DW according to the FRAP method. The TPC and TFC showed a high correlation with the AA according to the ABTS and the FRAP methods. The Pearson correlation between the TFC and AA/ABTS has a value of r = 0.92. The TFC and AA/FRAP present a value of r = 0.94.

## 1. Introduction

Fruits, in addition to their nutritional components, have phytocomponents that have biological properties and can exert a beneficial effect on certain systems in the human body. Diet is known to be an important factor in preventing cardiovascular disease and some cancers, Alzheimer’s disease, and other neurodegenerative diseases. The antioxidant activity is one of the biological activities of the phytocomponents of fruits most studied [1,2,3,4,5]. Natural antioxidants are compounds that can be found in food and can prevent the oxidation of other molecules. Oxidation is generated because of the action of free radicals and other reactive oxygen species, causing oxidative damage to cellular structures, DNA, proteins, and other biomolecules [6,7,8,9].

From phytochemical extractions and analysis of the phenolic profiles in fruits, it has been determined that the concentration of phenolic compounds varies depending on genetics (genus, species, cultivar, and genotype), environmental factors, and the state of maturity [10,11]. The genetic factor is a factor that influences this characteristic since the main propagation method of blackberry plants is by sexual seeds, which implies genetic segregation [12].

The Andean blackberry fruit (*Rubus glaucus* Benth) is a native plant of the Andean region of South America belonging to the *Rosacea* family. This plant is widely cultivated in countries such as Ecuador, Costa Rica, and Colombia. The Andean blackberry has been cultivated in other tropical countries. The fruits present small drupes with a size of around 1.0 to 2.5 cm. The mature fruits are of dark red or purple color and have a good aroma and bittersweet flavor [13,14,15]. The fruits of the *Rubus* genus are considered nutritious and healthy foods for their biological properties. The large number of species and the genetic variability of the genus Rubus, with the intervention of nature and man, have led to the generation of a wide type of cultivars and hybrids with different characteristics [16]. The blackberry (*Rubus* sp.) has been studied as a good source of antioxidants for its significant levels of phenolic compounds, such as ellagic acid, tannins, ellagitannins, quercetin, gallic acid, anthocyanins, and cyanidins [17,18,19,20,21,22].

Some studies suggest that the chemical structures of anthocyanins have an important influence on their biological activity; for example, mono-glycosylated and non-acylated anthocyanins are strong inhibitors of the growth and proliferation of colon cancer cells [23]. Häkkinen (2000) [24] mentions that the antioxidant and anti-carcinogenic effects of the fruits are attributable to the presence of phenolic acids and flavonoids present in the fruits. Garzón, Riedl, and Schwartz (2009) [25] have described the total polyphenol content (TPC) and anthocyanins in *Rubus glaucus* Benth as having values of 294 mg GAE/100 g FW and 45 mg/100g FW, respectively. They have also described the antioxidant activity of blackberry extracts using the ABTS and ferric reducing power (FRAP) methods with values of 2.01 and 4.50 mmol TE/100 g FW, respectively. Horvitz, Chanaguano, and Arozarena, (2017) [26] described the anthocyanin content and the TPC effects for maturity stages and storage conditions in Castilla Andean blackberries (*Rubus glaucus* Benth) grown in Tungurahua, Ecuador. They reported TPC with values between 446.00 and 736.00 mg GA/100 g DW depending on the maturity stage, temperature, and days of storage.

In Ecuador, there are four commercial varieties of blackberry (Castilla, Andimora, Colombiana, and Brazos) that are cultivated [27]. The blackberry of Castilla (*Rubus glaucus* Benth) is a fruit native to the tropical zones of America, mainly Colombia and Ecuador, and is distinguished by its dark red color and characteristic flavor [28]. In Ecuador, blackberry cultivation takes place in the valleys of the inter-Andean alley and in the foothills of the Sierra, in the provinces of Tungurahua, Cotopaxi, Imbabura, Pichincha, Chimborazo, and Bolívar [29]. The INIAP Andimora 2013 variety of blackberries without thorns comes from a mutation of a sexual blackberry from Castilla with thorns and a process of improvement by selection [30]; its fruit has a high content of soluble solids and vitamin C. The Colombiana cultivar also comes from a natural mutation and was produced by a selection process from wild blackberry plants; however, it presents a high variability of the species depending on the size, color, and quality of the fruit [31]. The Brazos blackberry (*Rubus* sp.) is a different species of blackberry obtained by crossing high-quality hybrids that gave rise to a large fruit but with a lower content of soluble solids [32].

The objective of this study was to evaluate the effect of the ripeness state of the fruits on the contents of TPC, total flavonoid content (TFC), and total anthocyanin content (TAC) in four blackberry cultivars, to establish the antioxidant activity at the different states of maturity for each cultivar.

## 2. Materials and Methods

### 2.1. Chemical Reagents

Deionized water obtained through a Milli-Q Academic water purification system (Millipore, Sao Paulo, Brazil) was used. The standards of (+) catechin, gallic acid, cyanidin-3-glucoside chloride, ABTS (2,2-azinobis-3-ethyl-benzothiazoline-6-sulfonic acid), and Trolox (6-hydroxy-2,5,7, 8-tetramethylchroman-2-carboxylic acid) used in the study were obtained from Sigma Aldrich (St. Louis, MO, USA). Analytical grade solvents and reagents were obtained from Merck (Darmstadt, Germany).

### 2.2. Plant Material

**Figure 1:**
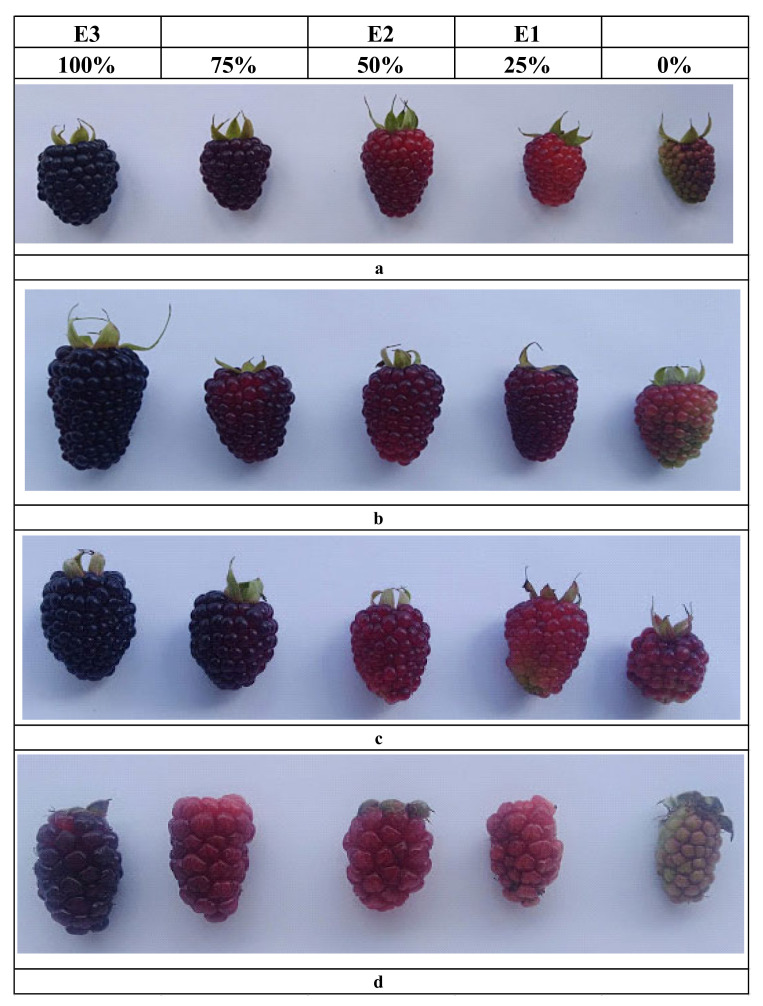
Photographs of blackberry (Rubus glaucus Benth) cultivars from Ecuador in different stages of maturity: (**a**) Blackberry Castilla variety, (**b**) Blackberry INIAP Andimora-2013 variety, (**c**) Blackberry Colombiana variety, and (**d**) Blackberry Brazos variety.

Four blackberry cultivars were used (Blackberry Castilla, Blackberry INIAP Andimora 2013, Blackberry Colombiana, and Blackberry Brazos) from the germplasm bank of the National Institute of Agricultural Research (INIAP), Quito, Ecuador. A total of 900 g of fruit was collected from the four blackberry cultivars (Figure 1). The fruit was harvested at three stages of maturity, based on a subjective indicator of covering color based on the degrees of maturity established in the Normas Técnicas-Instituto Ecuatoriano de Normalización (NTE INEN) 2427: 2016 standard [33]. The first stage of maturity (E1) was represented by a 25% color change in the covering, where the berries contain mostly green drupes and a few red or pink ones; the second (E2), by a 50% change in the covering color, where the largest number of drupes are deep red and a few are purple; and the third state (E3), by a 100% change in the covering color with purple drupes in its entirety, almost black (Figure 1). 

### 2.3. Preparation of Samples 

The fruits in their different stages of maturity were separated into two parts. The physicochemical characterization was carried out in the first portion in the fresh state; the second portion was packed into plastic bags with closure and stored in a freezer (−12 °C) for subsequent drying by lyophilization. The dried samples were subject to a grinding process in a Retsch model ZM 200 mill (Hann, Germany) then passed through a stainless-steel sieve (1 mm mesh) to ensure a uniform particle size. Finally, the samples were packed in plastic containers with hermetic lids, labeled, and protected from light until the time of carrying out the analysis.

### 2.4. Physicochemical Analysis

#### 2.4.1. Evaluation of Color 

The color of the berries was determined with a ColorTec-PCM handheld colorimeter (ColorTec, Clinton, NJ, USA), with a measurement angle of 10°, Illuminator D65, and aperture of 8 mm. The chromatic properties of each of the blackberry cultivars were determined using the L* a* b* color method of the CIE (Commission Internationale de l’Eclairage) and were expressed in terms of the L * lightness, a* red/green, and b* blue/yellow coordinates. An amount of 200 g of each cultivar was homogenized in a blender. A volume of 30 mL of each sample was placed in a Petri dish, avoiding the formation of lumps or bubbles. The Petri dish was placed on a white surface and divided into four equal parts. Measurements were performed in triplicate in each quarter and at the center of the plate [34].

#### 2.4.2. Determination of Total Soluble Solids (TSS)

The TSS concentration was determined by refractometry using an ATAGO digital refractometer (Tokyo, Japan), according to the methodology specified by AOAC 2000 [35]. Two drops of fruit juice were placed on the prism of the equipment surface, and the percentage of soluble solids was shown directly on the equipment screen, expressed in terms of °Brix.

#### 2.4.3. Determination of Titratable Acidity (TA)

The TA content of the blackberry cultivars was measured by potentiometric titration with the help of a standardized alkaline solution [35]. A total of 30 g of fruit pulp was weighed and washed with distilled water at a volume of 200 mL. Subsequently, 20 mL was placed in a 25 mL beaker and titrated with a 0.1 N NaOH solution until pH 8.2 was reached. The results are reported based on the predominant organic acid in the sample, in this case, citric acid.

#### 2.4.4. Maturity Index (MI)

The MI of the blackberry cultivars was determined using the relation between the content of total soluble solids (TSS) and the titratable acidity (TA) [35]. According to the equation (Equation (1)),

MI = TSS/TA(1)

where MI: Maturity Index, TSS: Total Soluble Solids (°Brix), and TA: Titratable Acidity (g citric acid/100g). 

### 2.5. Preparation of Extracts of Blackberries

The extraction of the phytocomponents was performed using the method proposed by Hue et al. (2014) [36]. An amount of 0.3 g of dry sample was placed in 15 mL plastic centrifuge tubes, and 5 mL of a methanol/water/formic acid solution (70:30:0.1 *v*/*v*/*v*) was added. The sample was subject to a shaking extraction process in a vortex (Mistral Multi-Mixer, Melrose Park, IL, USA) for 5 min and subsequently in an ultrasound bath (Cole-Palmer, Chicago, IL, USA) for 10 min. The sample was then centrifuged in a Damon IEC/Division centrifuge (Needham Hts., Needham, MA, USA) for 10 min at 5500 rpm (2706× g). The supernatant was separated and transferred to a 25 mL amber volumetric balloon. This process was repeated three more times and brought to volume with the extraction solution. The same extract was used for the antioxidant activity (AA) determination.

### 2.6. Quantification of Total Polyphenol Content (TPC)

TPC quantification was performed by UV-visible spectrophotometry using the method proposed by Samaniego et al. (2020) [37]. A volume of 1 mL of diluted extract was placed in a 15 mL test tube; 6 mL of distilled water and 1 mL of Folin–Ciocalteau reagent was added, and the mixture was left to rest for 3 min. Subsequently, 2 mL of 20% Na_2_CO_3_ (*w*/*v*) was added and heated at 40 °C for 2 min. This reaction formed a blue chromophore; its absorbance was measured at 760 nm on a Shimadzu model 2600 spectrophotometer (Shimadzu, Kyoto, Japan). The isolation of the compounds was performed with repeated cycles. Five extraction cycles were necessary to obtain 100% TPC recovery. The TPC quantification was performed by the interpolation of the corresponding absorbance of each sample on a calibration curve made with gallic acid at 0–100 mg gallic acid/L. The curve obtained was y = 0.0109x + 0.0618 (R^2^ = 0.999). The results are expressed as milligrams of gallic acid equivalents per gram of dry sample (mg GAE/g DW).

### 2.7. Quantification of Total Flavonoid Content (TFC)

TFC was determined by UV-visible spectrophotometry, using the method proposed by Zhishen et al. (1999) [38]. A volume of 1 mL of the diluted extract was placed in a 15 mL tube; 4 mL of distilled water was added, and the mixture was homogenized. Then, 0.3 mL of 5% sodium nitrite (*w*/*v*) and 0.3 mL of 10% aluminum chloride (*w*/*v*) were added consecutively, allowing the sample to stand for 5 min after the addition of each reagent. Finally, 2 mL of 1N NaOH was added, and the volume was made up to 10 mL with distilled water. In this reaction a pink chromophore was formed; its absorbance was measured at 490 nm with a Shimadzu model 2600 spectrophotometer (Shimadzu, Kyoto, Japan). The recovery of compounds was performed with repeated cycles. Five extraction cycles were necessary to obtain 100% TFC recovery. The quantification was performed by the interpolation of the corresponding absorbance of each sample on a calibration curve made with (+) - catechin 0–100 100 mg (+) - catechin/L. The curve obtained was y = 0.0022x + 0.0725 (R^2^ = 0.997). The results are expressed as milligrams of catechin equivalents per gram of dry sample (mg cat/g DW).

### 2.8. Quantification of Total Anthocyanin Content (TAC)

TAC was determined by UV-visible spectrophotometry, following the pH differential methodology proposed by Rapisarda et al. (2000) [39]. An amount of 0.3 g of lyophilized sample was placed in a 15 mL centrifuge tube; 5 mL of pH 1.0 buffer (KCl 0.2 N and HCl 0.2 N) was added and stirred in a Mistral Multi-Mixer vortex (Melrose Park, IL, USA) for 5 min then in a Cole-Parmer model 8892 ultrasound bath (Chicago, IL, USA) and finally centrifuged at 5500 rpm for 10 min. The supernatant was separated and transferred to a 25 mL amber volumetric balloon. This procedure was repeated three more times, and the mixture was brought to volume with the pH 1.0 buffer solution. Extraction with pH 4.5 buffer (CH_3_COONa 1M and HCl 1N) was performed using the same methodology. The anthocyanin quantification was performed by measuring the absorbance in the extracts (pH 1.0 and 4.5) at two wavelengths (510 and 700 nm) on a Shimadzu model 2600 spectrophotometer (Shimadzu, Kyoto, Japan). The recovery of the compounds was performed with repeated cycles. Seven extraction cycles were necessary to obtain 100% TAC recovery. The TAC was calculated by the absorbance differences between pH 1.0 and pH 4.5, as described in the equation (Equation (2)):

A = [(A510 − A700)pH1.0 − (A510 − A700 )pH4.5](2)

The TAC results are expressed as milligrams of cyanidin-3-glucoside chloride per gram of dry sample (mg cy-3-glu/g DW).

### 2.9. Evaluation of Antioxidant Activity by the ABTS Method

Antioxidant activity (AA) was evaluated by the 2,2-azinobis (3-ethyl-benzothiazoline-6-sulfonic acid) cation bleaching method (ABTS^•+^) following the methodology described by Zambrano et al. (2020) [40]. The ABTS^•+^ solution (7 mM) and the potassium persulfate solution (2.45 mM) were mixed in a 1:1 ratio (*v*/*v*). The next day, the absorbance of the previously prepared ABTS^•+^ working solution was measured, and it was diluted with phosphate buffer until obtaining an absorbance of 1.1 ± 0.01 at 734 nm. Volumes of 200 µL of the samples were placed in 15 mL test tubes, and 3.8 mL of the ABTS^•+^ working solution was added. The solution was left to stand for 45 min, and the final absorbance at 734 nm was measured using a Shimadzu model 2600 spectrophotometer (Shimadzu, Kyoto, Japan). The AA of the sample was obtained by interpolating the absorbance in a calibration curve previously elaborated with a Trolox standard (0–800 µmol Trolox/L). The curve obtained was y = 0.0014x + 0.0838 (R^2^ = 0.993). The results are reported as micromoles of equivalent Trolox per gram of dry sample (µmol TE/g of sample DW).

### 2.10. Evaluation of Antioxidant Activity by Ferric Reducing Power (FRAP) Method

The AA of the extracts was measured by the ferric reducing power (FRAP) method following the methodology described by Babu, Gurumurthy, Borra, and Cherian (2013) [41]. A volume of 1.0 mL of diluted extract was placed in a 15 mL test tube; 2.5 mL of phosphate buffer at pH 6.6 and 2.5 mL of a 1.0% potassium ferrocyanide solution were added. The mixture was shaken and incubated at 50 °C for 20 min. Subsequently, 2.5 mL of 10% trichloroacetic acid was added along with 2.5 mL of water and 0.5 mL of 1% FeCl_3_. The mixture was homogenized in a vortex (Mistral Multi-Mixer, Melrose Park, IL, USA). Finally, it was left to stand for 30 min in the dark; at this point, a green complex (ferrous chloride–potassium ferrocyanide) was formed, and the absorbance at 700 nm was measured in a Shimadzu model 2600 spectrophotometer (Shimadzu, Kyoto, Japan). The AA of the sample was obtained by the interpolation of the absorbance in a calibration curve previously elaborated with a Trolox standard of 0–800 µmol Trolox/L. The curve obtained was y = 0.0014x + 0.1524, (R^2^ =0.994). The results are expressed as micromoles of equivalent Trolox per gram of dry sample (DW) (µmol TE/g of sample DW).

### 2.11. Statistical Analysis

The results are reported as the mean ± standard deviation (SD) (*n = 3*) of each parameter evaluated in the samples of each cultivar in its respective state of maturity. The investigation was carried out using a completely randomized design in a 4 × 3 factorial arrangement, with 3 repetitions per treatment (*n = 36*). ANOVA one-way analysis of variance was performed to determine significant statistical differences between cultivars and ripening stages. Tukey’s test was used at 5% to determine differences between the means of each treatment. Statistical analysis was performed using the Statistica 10.0 software for Windows (Statsoft, Paris, France).

## 3. Results and Discussion

### 3.1. Physicochemical Analysis

#### 3.1.1. Determination of Color

Physicochemical characterization of the fruits was carried out in order to evaluate their state of maturity and their relationship with color, since the sampling was carried out in a subjective way (visually) according to the color table of fruit coating [33]. Kalt et al. (1995) [42] have described that the color is related to the state of maturity due to the accumulation of pigments and the variation of the content of sugars and organic acids in the fruits. Table 1 shows the color coordinate values determined by the CIE L* a* b* method.

**Figure 2:**
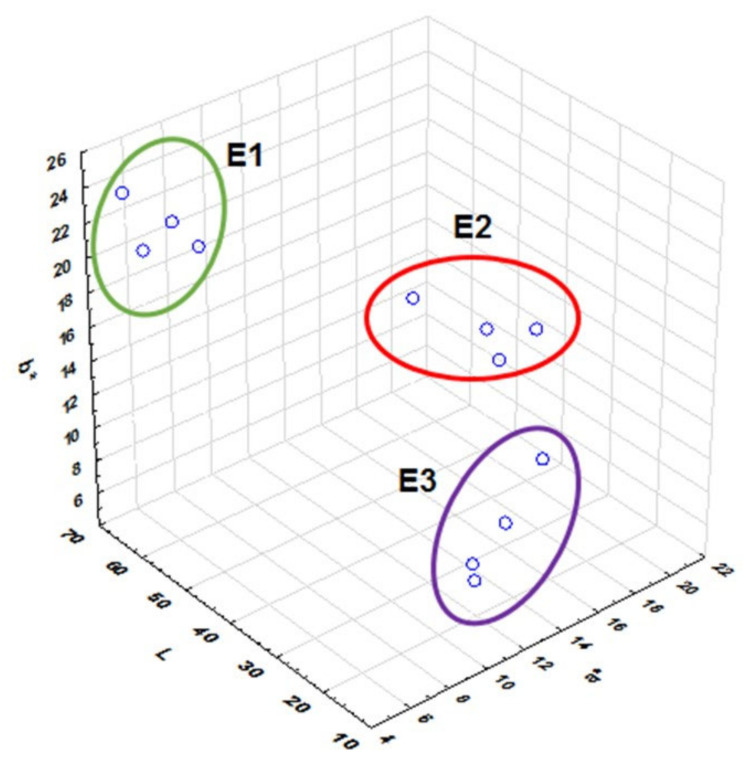
Color coordinates (method CIE L*a*b*) of four blackberry cultivars for three-maturity-stage fruits.E1 (E1-25%), E2 (E2-50%) and E3 (E3-100%).

The four blackberry cultivars show high variability in the colorimetric coordinates a* (red) and b* (blue). These elements, together, contribute to the hue of these berries. The values of lightness (L*) define the intensity of the colors of the samples; the lightness of the fruit pulp tends to decrease as its maturation progresses [34]. For example, for the Colombiana variety, the values (L*) were E1-25% (66.87 ± 2.92), E2-50% (45.07 ± 3.57), and E3-100% (19.73 ± 0.59). The color changed from a yellow trend in the E1—25% state (high values of b* between 19.60 ± 6.83 and 24.04 ± 0.83) to a reddish hue in the E2-50% state (values of a* between 16.74 ± 2.15 and 20.56 ± 0.82) and ends with a purple hue in the state of maturity E3-100% (values of a* between 11.78 ± 0.75 and 17.87 ± 2.56 and values of b* between 6.29 ± 2.83 and 8.58 ± 1.83). This trend agrees with the trends reported by Tosun, Ustun, and Tekguler (2008) [43]. These results confirm that the state of maturity of the samples is related to the color of the fruit; when the ripening process progresses, it tends to produce a reddish to purple coloration, due to the accumulation of anthocyanins in the fruits (Figure 2).

Llerena et al. (2019) [34] described the color analysis in three fruits grown in Ecuador (tree tomato, naranjilla, and arazá). They found a correlation between the color and the content of phytocompounds such as polyphenols, anthocyanins, and carotenoids. When the green color attributed to the degradation of the chlorophyll structure is lost, the biosynthesis of pigments such as carotenoids and anthocyanins takes place conferring the orange, red, and violet colorations [44]. In Figure 2, the distinction of the three-color tones according to the state of maturity can be observed, confirming that the samples were collected correctly, allowing a study of the content of phytocomponents during the maturation process.

#### 3.1.2. Total Soluble Solids (TSS), Titratable Acidity (TA), and Maturity Index (MI)

In Table 2, the results of the TSS and TA determination are presented. The maturity index was established and compared to the color status of the coating (E1-25%, E2-50%, and E3-100%) of the fruits of the four cultivars. It was observed that as the color of the fruit changed from yellow to purple during the ripening process, the sugar content increased in the different stages of maturity and the acidity decreased. For example, TSS increases in content for the blackberry Brazos cultivar for E1-25% (6.32 ± 0.13 °Brix), E2-50% (7.07 ± 0.03 °Brix), and E3-100% (9.81 ± 0.08 °Brix), while TA decreases in the four cultivars. For the cultivar Brazos, the TA values for E1-25% (3.56 ± 0.04%), E2-50% (2.68 ± 0.03%), and E3-100% (1.58 ± 0.03%) were found. Farinango (2010) describes this same phenomenon in his study with an increase in TSS and a decrease in TA as the fruit matures. The TSS increased from 6.00 ± 0.39 to 8.62 ± 0.50 °Brix for the Brazos variety, and an increase from 7.78 ± 0.37 to 11.30 ± 0.68 °Brix for the cultivar Castilla was observed. The TA exhibited a decrease (2.46 ± 0.19 to 1.61 ± 0.10) for the Brazos variety [45].

Horvitz, Chanaguano, and Arozarena, (2017) described the same behavior for the commercial blackberry variety of Castilla—an increase in TSS and a decrease in TA as the fruit matures, with values of TSS (9.63 ± 0.69 to 11.00 ± 1.67 °Brix) and TA (3.80 ± 0.05 to 2.78 ± 0.21%) for the maturity stages 3 and 5, respectively [26]. The analysis of variance (ANOVA) showed that there is an effect of the cultivar and the state of maturity on the content of TSS and TA (*p* < 0.05).

The correlation analysis between the MI obtained in the laboratory (TSS/TA ratio) and the state of maturity at which the fruit was harvested (defined by the color change in the covering) allowed establishing that there is a high correlation between these two parameters. The correlation coefficients were (r2) 0.989, 0.994, 0.987, and 0.995 for the blackberry Brazos, Colombiana, Castilla, and Andimora cultivars, respectively. These values demonstrate that the samples were harvested at the appropriate maturity stage to evaluate the content of antioxidants during the maturation process of the four blackberry cultivars.

The Ecuadorian standard NTE INEN 2427:2016 [33] establishes the minimum limits for TSS and maximum limits for TA for commercial blackberry varieties from Castilla and Brazos. The minimum TSS values were 9.0 °Brix for the Castilla variety and 7.0 °Brix for the Brazos variety. The maximum allowed TA values for the Castilla variety are 2.70% and 2.10% citric acid for the blackberry Brazos variety. The results obtained in this TSS and TA study of the blackberry Castilla and Brazos varieties are in accordance with the Ecuadorian INEN standard. In the case of the blackberry Andinamora cultivar, the results obtained in this study are comparable to those reported by Brito et al. (2016) [46] and Llerena et al. (2019) [34]. They reported that during maturation, the TSS of the fruit increased up to 12.60 and 12.69 °Brix, respectively, while the TA decreased to 2.62 and 2.81% citric acid, respectively. On the other hand, the TSS contents obtained in this study are higher than those determined by Vasco et al. (2008) [47] for the cultivar of Castilla (11.00 °Brix).

### 3.2. Total Polyphenol Content (TPC)

Table 3 shows the TPC values of the four blackberry cultivars studied. The TPC content decreased during the maturation process of the four blackberries cultivars (Brazos, Colombiana, Castilla and Andimora). For example, for the Andimora variety, a variation from E1-25% (76.43 ± 5.98 mg GAE/g DW) to E3-100% (46.19 ± 1.02 mg GAE/g DW) was observed. The highest TPC content was observed in the E1-25% maturity stage. In this maturity stage, the Colombiana cultivar presented the highest TPC content (81.10 mg GAE/g DW), while Brazos exhibited the lowest value (51.26 mg GAE/g DW). However, in the E3-100% state, Andimora was observed to have the highest TPC content (46.19 mg GAE/g DW). In terms of percentage, the TPC content decreased by 38.37%, 44.29%, 19.45%, and 39.57% for the blackberry Brazos, Colombiana, Castilla, and Andimora cultivars, respectively.

The results obtained showed that the TPC content decreased significantly from the E1-25% state of maturity to E2-50% but slightly varied between the E2-50% and E3-100% states. The analysis of variance (ANOVA) of the results established that there is an effect of the state of maturity on the TPC content (*p* < 0.05); this effect is generated as a consequence of the biochemical reactions in the fruit, since phenolic acids are more abundant in the early stages of fruit development, that is, in green fruits. Wang and Lin (2000) [18] have described that the TPC decreases with increasing ripening, reflecting a higher content in the first state represented by the green color of the fruit. On the other hand, the analysis of variance (ANOVA) of the results established that there is an effect of the cultivar on the TPC content (*p* < 0.05), confirming the studies carried out by Bernal et al. (2014) who reported that the content of TPC varies in cultivars of the same genus Rubus. These differences are associated with the genetic characteristics of each cultivar and post-harvest conditions [11].

Additionally, the results obtained allowed it to be established that in the edible maturity stage, E3-100% of color change in coating, the TPC content was 31.59 ± 1.03, 45.18 ± 0.97, 44.68 ± 2.28, and 46.19 ± 1.02 mg GAE/g DW for the blackberry Brazos, Colombiana, Castilla, and Andimora cultivars, respectively. These results are within the range reported by Kaume et al. (2012) for the TPC content in both commercial and native blackberries, whose values vary between 1.14 and 10.56 mg GAE/g FW (9.56 and 89.76 mg GAE/g DW) [48] For the blackberry Castilla cultivar, the results obtained are within the range obtained by Mertz et al. (2009) [49] and Garzón et al. (2009) [25] for TPC in this cultivar (23.40 to 63.00 mg GAE/g DW). In the cultivar Andinamora, the results obtained are lower than those reported by Llerena et al. (2014) (63.52 mg GAE/g DW) [50].

### 3.3. Total Flavonoid Content (TFC)

Table 3 shows the blackberry cultivar TFC content results. The TFC content decreased with the ripening process of the fruits in the four cultivars tested. In the Andimora variety a variation from E1-25% (14.20 ± 0.79 mg cat/g DW) to E3-100% (10.75 ± 0.64 mg cat/g DW) was observed. In the mature state E2-25% (coating color change), the highest TFC content was obtained in the four blackberry cultivars. In this state of maturity, the Colombiana cultivar presented the highest TFC content with 19.15 ± 0.77 mg cat/g DW, followed by blackberry Andimora with 14.20 ± 0.79, blackberry Brazos with 13.76 ± 0.11, and blackberry Castilla with 11.19 ± 0.94 mg cat/g DW. The TFC content decreased by 41.71%, 35.77%, 9.65%, and 24.30% for the blackberry Brazos, Colombiana, Castilla, and Andimora cultivars, respectively.

In the state of complete maturity E3 (100% change of color in the coating), TFC contents of 12.30 ± 0.63, 10.75 ± 0.64, 10.11 ± 0.14, and 8.02 ± 0.50 mg Cat/g DW were obtained for the blackberry Colombiana, Andinamora, Castilla, and Brazos cultivars, respectively (Table 3). These results are similar to those obtained by Armijos (2018), who reported that the TFC content in the Andean blackberry from three locations in Ecuador varied within the range of 1.20 to 1.35 mg cat/g FW (12.00 to 13.50 mg cat/g DW) [51]. The analysis of variance (ANOVA) of the results established that there is an effect of the state of maturity and cultivar on the content of total flavonoids (*p* < 0.05), confirming that TFCs tend to decrease as the state of fruit maturity progresses due to the balance between biosynthesis and metabolism. Flavonoids are, in turn, in lower concentrations since they are part of the TPC, and their content also varies depending on the type of cultivar.

### 3.4. Total Anthocyanin Content (TAC)

Table 3 shows the blackberry cultivars’ TAC content. The TAC content increased during the maturation process. For the Brazos blackberry cultivar, the TAC content increased from 0.67 ± 0.50 to 8.63 ± 12.59 mg cy-3-glu/g DW; for the blackberry Colombiana cultivar, from 0.40 ± 2.29 to 12.26 ± 53.05 mg cy-3-glu/ g DW; for the blackberry cultivar of Castilla, from 1.02 ± 7.50 to 10.89 ± 104.23 mg cy-3-glu/g DW; and for the blackberry cultivar of Andimora, from 1.11 ± 2.32 to 9.26 ± 28.81 mg cy-3-glu/g DW. At the E3-100% maturity stage (100% coating color turn), the blackberry Colombiana cultivar exhibited the highest TAC content (12.26 ± 53.05 mg cy-3-glu/g DW), while the blackberry Brazos cultivar showed the lowest value (8.63 ± 12.59 mg cy-3-glu/g DW). The analysis of variance (ANOVA) of the results established that there is an effect of the state of maturity and cultivar on the TAC content (*p* < 0.05). These results confirm studies reported by Häkkinen (2000). These types of biomolecules are synthesized during the ripening process; in the case of red fruits, an accumulation of anthocyanins occurs at the end of their maturity stage [24]. The TAC concentration obtained in the four cultivars at the stage of full maturity E3-100% was much higher than that obtained by Garzón et al. (2009) [25] in the species *Rubus glaucus* and Ivanovic et al. (2014) with the species Rubus fruticosus, corresponding to 3.79 and 1.15 mg cy-3-glu/g DW, respectively [52]. On the other hand, the Andimora cultivar in this mature state had an TAC content of 9.26 ± 28.81 mg cy-3-glu/g DW, a lower result than that reported by Llerena et al. (2014) (14.16 ± 158.71 mg cy-3-glu/g DW) [50].

### 3.5. Antioxidant Activity (AA) 

#### 3.5.1. Evaluation of Antioxidant Activity by the ABTS Method

The results obtained in the AA evaluation by the ABTS method for the four blackberry cultivars at three different stages of maturity are presented in Table 4. The results show that, during the ripening of the fruit, the AA decreased in the four blackberry cultivars. For example, the AA decreased from E1-25% (630.61 ± 20.66 µmol TE/g DW) to E3-100% (344.42 ± 4.05 µmol TE/g DW) for the blackberry Brazos cultivar. This decrease represented 45.38% in the Brazos cultivar, 54.44% in the Colombiana cultivar, 6.18% in the Castilla cultivar, and 36.21% in the Andimora cultivar. The blackberry Colombiana and Andimora cultivars showed the highest AA in the E2-50% state at 1278.63 ± 141.14 and 929.30 ± 40.95 µmol TE/g DW, respectively. In the fully mature stage, the cultivars of Castilla and Andinamora presented the highest values, (658.28 ± 31.30 and 592.76 ± 25.37 µmol TE/g DW, respectively).

The results of statistical analysis show that there is an effect of the state of maturity and cultivar on AA (*p* < 0.05), confirming that there is a significant decrease in AA during fruit ripening in the four cultivars. These results agree with those reported by Garzón et al. (2009) [25], who reported that there is a decrease in AA for the Brazos blackberry cultivar from 711.91 to 531.62 µmol TE/g DW and from 762.02 to 559.25 µmol TE/g DW for blackberries from Castilla during the ripening process. In E3-100%, the AA value obtained by the ABTS method for the cultivar of Castilla (658.28 ± 31.30 µmol TE/g DW) was higher than that reported by Vasco et al. (2008) [47] and similar to that obtained by Garzón et al. (2009) [25], which were 464.10 µmol TE/g DW and 559.25 µmol TE/g DW, respectively.

#### 3.5.2. Evaluation of Antioxidant Activity by the FRAP Method

Table 4 shows the results for the AA evaluated by the ferric reducing power (FRAP) method for the four blackberry cultivars. The AA values measured using the FRAP method decreased during fruit ripening in the four cultivars studied. For example, the AA decreased from 717.13 ± 43.18 to 338.43 ± 28.85 µmol TE/g DW in the Brazos cultivar. This decrease represented 52.81% for the Brazos cultivar, 56.16% for the Colombiana cultivar, 28.90% for the Castilla cultivar, and 41.70% for the Andimora cultivar of antioxidant loss. The analysis of variance (ANOVA) of the results established that there is an effect of the state of maturity and cultivar on the AA according to the FRAP method (*p* < 0.05).

The results obtained for E3-100% in the cultivars of *Rubus glaucus* Benth were higher than those obtained by Schulz et al. (2019) [53] in *Rubus ulmifolius*, who obtained 241.06 μmol Fe ^+2^/g DW, and lower than those obtained by Armijos (2018) [51], who reported AA values according to ABTS of 98.23 to 109.45 µmol Trolox/g FW ( 884.07 to 985.05 µmol Trolox/g DW), in Andean blackberries from different locations in Ecuador. Vasco et al. (2008) [47] reported AA values according to this method of 6.2 mmol Trolox/100g FW (corresponding to 523.21 µmol Trolox/g DW) for the blackberry of Castilla (*Rubus glaucus* Benth) in its mature edible state. This is a value similar to those obtained in the present investigation for the state E3-100% for the blackberry of Castilla (548.23 ± 45.29 µmol TE/g DW) and blackberry Colombiana cultivar (563.08 ± 6.48 µmol TE/g DW) and lower than that obtained for the blackberry Andimora cultivar (655.43 ± 29.17 µmol TE/g DW).

Finally, a Pearson correlation analysis was performed with the antioxidant activity data measured by the ABTS and FRAP methods of the four blackberry cultivars at the three stages of maturity. The Pearson correlation analysis shows a significant correlation between TPC and AA/ABTS with r = 0.86, and TFC and AA/ABTS with r = 0.92. Significant correlations of TPC (r = 0.94) and TFC (r = 0.88) with AA/FRAP were observed. The blackberry cultivars’ AAs have good correlation with the TPC and TFC components.

## 4. Conclusions 

The state of the maturity of the fruits is used as a quality criterion for the products reaching the consumer. The state of maturity can also be used to predict the possible content of secondary metabolites with antioxidant capacity such as polyphenols, flavonoids, and anthocyanins. In the four blackberry cultivars tested, as the maturity stage advanced, there was a reduction in the content of phytochemical components and a reduction in the antioxidant activity. As the blackberries of the four cultivars matured, the TSS content increased and the TA content decreased. The highest contents of TPC, TFC, and AA were observed in the E1-25% state, which constitutes an initial stage of maturation. However, the fruit is not edible in this state.

The blackberry cultivars presenting the highest AA were the Colombiana and Andimora blackberry cultivars; both correspond to the species Rubus glaucus (without spines) and descendants of the blackberry cultivar from Castilla. The TAC content increased significantly during the maturation process until reaching its maximum in the E3-100% state (full maturation), and the Colombiana blackberry and Castilla blackberry cultivars stood out. These results indicate that including fruits such as blackberries in the diet may allow access to significant amounts of compounds with antioxidant capacity. This could have a preventive effect against certain diseases. Blackberries can be used to develop natural extracts with antioxidant capacity and be used in the food industry.

## Figures and Tables

**Table 1:** Commission Internationale de l’Eclairage (CIE) (L* a* b*) color coordinates in four blackberry cultivars grown in Ecuador.

Cultivar	Maturity Stage	Color				
		L*	a*	b*	C*	°H
		Lightness	(+ Red, − Green)	(+ Yellow, − Blue)	Chroma	Hue
Brazos	E1-25%	65.76 ± 2.27	4.98 ± 0.81	24.04 ± 0.83	24.55 ± 0.98	11.67 ± 1.48
	E2-50%	52.50 ± 3.20	16.74 ± 2.15	14.57 ± 5.83	22.38 ± 0.67	49.00 ± 7.02
	E3-100%	20.85 ± 0.60	12.05 ± 0.10	6.97 ± 0.98	13.93 ± 2.58	59.99 ± 4.47
Colombiana	E1-25%	66.87 ± 2.92	6.11 ± 2.19	19.94 ± 2.83	20.96 ± 0.47	17.20 ± 8.19
	E2-50%	45.07 ± 3.57	19.66 ± 0.95	10.79 ± 4.83	22.45 ± 0.65	61.20 ± 8.97
	E3-100%	19.73 ± 0.59	11.78 ± 0.75	6.29 ± 2.83	13.37 ± 0.45	61.88 ± 3.21
Castilla	E1-25%	64.52 ± 2.46	7.12 ± 1.66	21.58 ± 5.83	22.77 ± 1.06	18.31 ± 0.79
	E2-50%	40.84 ± 4.08	20.56 ± 0.82	12.89 ± 3.85	24.27 ± 1.40	57.93 ± 1.28
	E3-100%	23.95 ± 1.48	14.60 ±1.83	7.45 ± 2.83	16.39 ± 0.22	62.97 ± 1.85
	E1-25%	64.21 ± 2.56	8.39 ± 2.71	19.60 ± 6.83	21.47 ± 0.68	23.23 ± 2.76
Andinamora	E2-50%	50.49 ± 4.25	20.39 ± 1.06	11.32 ± 3.83	23.33 ± 2.09	61.00 ± 1.28
	E3-100%	29.01 ± 3.80	17.87 ± 2.56	8.58 ± 1.83	19.82 ± 2.84	64.34 ± 0.77

Results are expressed as mean ± standard deviation (SD) (*n = 3*).

**Table 2:** Physicochemical analysis of four blackberry cultivars.

Cultivar	Maturity Stage	Soluble Solids (°Brix)		Titratable Acidity (% Citric Acid)		Maturity Index (TSS/TA)	
Brazos	E1-25%	6.32 ± 0.13	Cg	3.56 ± 0.04	Acd	1.77 ± 0.05	Cd
	E2-50%	7.07 ± 0.03	Bf	2.68 ± 0.03	Bf	2.64 ± 0.02	Bc
	E3-100%	9.81 ± 0.08	Ac	1.58 ± 0.03	Cg	6.21 ± 0.18	Aa
Colombiana	E1-25%	6.23 ± 0.19	Cg	3.98 ± 0.11	Abc	1.57 ± 0.08	Cde
	E2-50%	8.36 ± 0.250	Bd	3.25 ± 0.10	Bde	2.57 ± 0.13	Bc
	E3-100%	11.00 ± 0.131	Ab	2.82 ± 0.11	Cef	3.91 ± 0.18	Ab
Castilla	E1-25%	6.08 ± 0.08	Cg	5.28 ± 0.15	Aa	1.15 ± 0,03	Ce
	E2-50%	7.64 ± 0.08	Be	4.27 ± 0.23	Bb	1.79 ± 0.10	Bd
	E3-100%	12.26 ± 0.31	Aa	3.19 ± 0.19	Cde	3.86 ± 0.30	Ab
Andinamora	E1-25%	6.19 ± 0.12	Cg	4.95 ± 0.26	Aa	1.25 ± 0.05	Ce
	E2-50%	7.47 ± 0.18	Bef	4.23 ± 0.23	Bb	1.77 ± 0.10	Bd
	E3-100%	12.14 ± 0.12	Aa	2.96 ± 0.27	Cef	4.12 ± 0.33	Ab

Results are expressed as mean ± standard deviation (SD) (*n* = 3). Capital letters indicate significant differences between states of maturity (*p* < 0.05). Lowercase letters indicate significant differences found between cultivars (*p* < 0.05) using the ANOVA one-way analysis followed by Tukey’s test.

**Table 3:** Effect of maturity stage on total polyphenol content (TPC), total flavonoid content (TFC), and total anthocyanin content (TAC) of four blackberry cultivars of Ecuador.

Cultivar	Maturity Stage	TPC (mg GAE/g DW)		TFC (mg Cat/g DW)		TAC (mg cy-3-glu/g DW)	
Brazos	E1-25%	51.26 ± 2.46	Abcd	13.76 ± 0.11	Abc	0.67 ± 0.50	Cf
	E2-50%	38.16 ± 1.64	Bfg	11.19 ± 0.21	Bdef	1.21 ± 4.29	Bef
	E3-100%	31.59 ± 1.03	Bg	8.02 ± 0.50	Bg	8.63 ± 12.19	Ac
Colombiana	E1-25%	81.10 ± 4.44	Aa	19.15 ± 0.77	Aa	1.40 ± 2.29	Cef
	E2-50%	52.51 ± 2.92	Bbce	12.64 ± 1.00	Bbcf	2.95 ± 5.13	Bd
	E3-100%	45.18 ± 0.97	Bdef	12.30 ± 0.63	Bcdf	12.26 ± 5.30	Aa
Castilla	E1-25%	55.47 ± 1.34	Ab	11.19 ± 0.94	Adef	1.02 ± 7.50	Cf
	E2-50%	42.89 ± 2.16	Bef	10.89 ± 0.94	Bdef	2.55. ± 20.95	Bd
	E3-100%	44.68 ± 2.28	Bdef	10.11 ± 0.14	Be	10.89 ± 10.23	Ab
Andimora	E1-25%	76.43 ± 3.98	Aa	14.20 ± 0.79	Ab	1.11 ± 2.32	Cef
	E2-50%	42.86 ± 1.60	Bef	12.59 ± 0.24	Bbcf	2.16 ± 1.62	Bde
	E3-100%	46.19 ± 1.02	Bcde	10.75 ± 0.64	Bde	9.26 ± 28.81	Ac

Results are expressed as mean ± standard deviation (SD) (*n = 3*). Capital letters indicate significant differences between states of maturity (*p* < 0.05). Lowercase letters indicate significant differences found between cultivars (*p* < 0.05) using the ANOVA one-way analysis followed by Tukey’s test.

**Table 4:** Antioxidant activity of four blackberry cultivars from Ecuador.

		Antioxidant Activity			
Cultivar	Maturity Stage	ABTS (µmol TE/g DW)		FRAP (µmol TE/g DW)	
Brazos	E1-25%	630.61 ± 20.66	Ad	717.13 ± 43.18	Acd
	E2-50%	550.67 ± 39.66	Bd	546.03 ± 21.33	Be
	E3-100%	344.42 ± 4.05	Be	338.43 ± 28.85	Cf
Colombiana	E1-25%	1278.63 ± 14.14	Aa	1284.55 ± 62.80	Aa
	E2-50%	793.88 ± 47.70	Bbc	825.29 ± 51.55	Bc
	E3-100%	582.59 ± 23.45	Bd	563.08 ± 6.48	Ce
Castilla	E1-25%	701.62 ± 38.50	Acd	771.05 ± 34.24	Ac
	E2-50%	660.40 ± 45.00	Bcd	721.92 ± 23.08	Bcd
	E3-100%	658.28 ± 31.30	Bcd	548.23 ± 45.30	Ce
Andimora	E1-25%	929.30 ± 40.95	Ab	1124.22 ± 60.33	Ab
	E2-50%	581.15 ± 8.12	Bd	795.29 ± 10.07	Bc
	E3-100%	592.76 ± 25.37	Bd	655.43 ± 29.17	Cde

Results are expressed as mean ± standard deviation (SD) (*n = 3*). Capital letters indicate significant differences between states of maturity (*p* < 0.05). Lowercase letters indicate significant differences found between cultivars (*p* < 0.05) using the ANOVA one-way analysis followed by Tukey’s test.

## References

[1] Capocasa F., Scalzo J., Mezzetti B., Battino M. (2008). Combining quality and antioxidant attributes in the strawberry: The role of genotype. Food Chem..

[2] Andersen Q.M., Markham K.R. (2006). Flavonoids, Chemistry, Biochemistry and Applications.

[3] Huang W., Zhang H., Liu W., Li C. (2012). Survey of antioxidant capacity and phenolic composition of blueberry, blackberry, and strawberry in Nanjing. J. Zhejiang Univ. Sci. B.

[4] Boeri P., Piñuel L., Dalzotto D., Monasterio R., Fontana A., Sharry S., Barrio D.A., Carrillo W. (2020). Argentine Patagonia barberry chemical composition and evaluation of its antioxidant capacity. J. Food Biochem..

[5] Piñuel L., Boeri P., Zubillaga F., Barrio D.A., Torreta J., Cruz A., Vásquez G., Pinto A., Carrillo W. (2019). Production of white, red and black quinoa (*Chenopodium quinoa* Willd Var. Real) protein isolates and its hydrolysates in germinated and non-germinated quinoa samples and antioxidant activity evaluation. Plants.

[6] Coronado H.M., Vega S., Gutiérrez T.R., Vázquez F.M., Radilla V.C. (2015). Antioxidants: Present perspective for the human health. Rev. Chil. Nutr..

[7] Vilcacundo R., Barrio D.A., Piñuel L., Boeri P., Tombari A., Pinto A., Welbaum J., Hernández-Ledesma B., Carrillo W. (2018). Inhibition of lipid peroxidation of kiwicha (*Amaranthus caudatus*) hydrolyzed protein using zebrafish larvae and embryos. Plants.

[8] Carrillo W., Gómez-Ruiz J.A., Miralles B., Ramos M., Barrio D., Recio I. (2016). Identification of antioxidant peptides of hen egg-white lysozyme and evaluation of inhibition of lipid peroxidation and cytotoxicity in the Zebrafish model. Eur. Food Res. Technol..

[9] Piñuel L., Vilcacundo E., Boeri P., Barrio D.A., Morales D., Pinto A., Morán R., Samaniego I., Carrillo W. (2019). Extraction of protein concentrate from red bean (*Phaseolus vulgaris* L.): Antioxidant activity and inhibition of lipid peroxidation. J. Appl. Pharm. Sci..

[10] Lee J., Dossett M., Finn C.E. (2012). Rubus fruit phenolic research: The good, the bad, and the confusing. Food Chem..

[11] Bernal L.J., Melo L.A., Díaz Moreno C. (2014). Evaluation of the antioxidant properties and aromatic profile during maturation of the blackberry (*Rubus glaucus* Benth) and the bilberry (*Vaccinium meridionale* Swartz). Rev. Fac. Nac. Agron..

[12] Vásquez W., Pupiales P., Viteri P., Sotomayor A., Feican C., Campaña D., Viera W. (2019). Chemical scarification and use of gibberellic acid for seed germination of blackberry cultivars (*Rubus glaucus* Benth). Interciencia.

[13] Meret M., Brat P., Mertz C., Lebrun M., Günata Z. (2011). Contribution to aroma potential of Andean blackberry (*Rubus glaucus* Benth.). Food Res. Int..

[14] Morales A.L., Albarracin D., Rodriguez J., Duque C., Riano L.E., Espitia J. (1996). Volatile constituents from Andes berry (*Rubus glaucus* Benth). J. High. Resolut. Chrom..

[15] Ramos F.A., Delgado J.L., Bautista E., Morales A.L., Duque C. (2005). Changes in volatiles with the application of progressive freeze-concentration to Andes berry (*Rubus glaucus* Benth). J. Food Eng..

[16] Bobinaitė R., Viškelis P., Venskutonis P.R. (2012). Variation of total phenolics, anthocyanins, ellagic acid and radical scavenging capacity in various raspberry (*Rubus* spp.) cultivars. Food Chem..

[17] Acosta-Montoya Ó., Vaillant F., Cozzano S., Mertz C., Pérez A.M., Castro M.V. (2010). Phenolic content and antioxidant capacity of tropical highland blackberry (*Rubus adenotrichus* Schltdl.) during three edible maturity stages. Food Chem..

[18] Wang S.Y., Lin H. (2000). Antioxidant activity in fruits and leaves of blackberry, raspberry, and strawberry varies with cultivar and developmental stage. J. Agric. Food Chem..

[19] Mertz C., Cheynier V., Günata Z., Brat P. (2007). Analysis of phenolic compounds in two blackberry species (*Rubus glaucus* and *Rubus adenotrichus*) by high-performance liquid chromatography with diode array detection and electrospray ion trap mass spectrometry. J. Agric. Food Chem..

[20] Vasco C., Riihinen K., Ruales J., Kamal-Eldin A. (2009). Phenolic compounds in *Rosaceae* fruits from Ecuador. J. Agric. Food Chem..

[21] Cuevas-Rodríguez E.O., Yousef G.G., García-Saucedo P.A., López-Medina J., Paredes-López O., Lila M.A. (2010). Characterization of anthocyanin and pro-anthocyanidins in wild and domesticated Mexican blackberries (*Rubus* spp.). J. Agric. Food Chem..

[22] Cuevas-Rodríguez E.O., Dia V.P., Yousef G.G., García-Saucedo P.A., López-Medina J., Paredes-Lopez O., González de Mejía E., Lila M.A. (2010). Inhibition of pro-inflammatory responses and antioxidant capacity of Mexican blackberry (*Rubus* spp.) extracts. J. Agric. Food Chem..

[23] Jing P., Bomser J.A., Schwartz S.J., He J., Magnuson B.A., Giusti M.M. (2008). Structure-function relationships of anthocyanins from various anthocyanin-rich extracts on the inhibition of colon cancer cell growth. J. Agric. Food Chem..

[24] Häkkinen S. (2000). Flavonols and Phenolic Acids in Berries and Berry Products. Ph.D. Thesis.

[25] Garzón G.A., Riedl K.M., Schwartz S.J. (2009). Determination of anthocyanins, total phenolic content, and antioxidant activity in Andes berry (*Rubus glaucus* Benth). J. Food Sci..

[26] Horvitz S., Chanaguano D., Arozarena I. (2017). Andean blackberries (*Rubus glaucus* Benth) quality as affected by harvest maturity and storage conditions. Sci. Hortic..

[27] Iza M., Viteri P., Hinojosa M., Martínez A., Sotomayor A., Viera W. (2020). Morphological, phenological and pomological differentiation of commercial cultivars of blackberry (*Rubus glaucus* Benth). Enfoque UTE.

[28] Rojas-Llanes J.P., Martínez J.R., Stashenko E.E. (2014). Content of phenolic compounds and antioxidant capacity of blackberry (Rubus glaucus Benth) extracts obtained under different conditions. Vitae.

[29] Jácome R., Ayala G., Martínez A., Viteri P., Vásquez W., Sotomayor A., Galarza D., Garcés S., Velásquez J., Sánchez V., Zambrano J. (2016). Caracterización del sistema de producción, zonas de producción y tipificación de productores del Ecuador. El Cultivo de Mora en el Ecuador.

[30] Viera W., Sotomayor A., Viteri P. (2019). Breeding of three Andean fruit crops in Ecuador. Chron. Hortic..

[31] Grijalba C., Calderón L., Pérez M. (2010). Yield and fruit quality of Andean blackberry (*Rubus glaucus* Benth), thorn and thornless, cultivated in open field conditions in Cajicá (Cundinamarca, Colombia). Rev. Fac. Cienc. Básicas.

[32] Rubio G. (2014). Investigación de la Mora y Propuesta Gastronómica. Bachelor’s Thesis.

[33] INEN. 2016. NTE INEN 2427. Frutas Frescas. Mora. http://181.112.149.204/buzon/normas/nte_inen_2427-1.pdf.

[34] Llerena W., Samaniego I., Angos I., Brito B., Ortiz B., Carrillo W. (2019). Biocompounds content prediction in ecuadorian fruits using a mathematical model. Foods.

[35] AOAC (2000). Official Methods of Analysis.

[36] Hue C., Brat P., Gunata Z., Samaniego I., Servent A., Morel G., Davrieux F. (2014). Near infra-red characterization of changes in flavan-3-ol derivatives in cocoa (*Theobroma cacao* L.) as a function of fermentation temperature. J. Agric. Food Chem..

[37] Samaniego I., Espin S., Cuesta X., Arias V., Rubio A., Llerena W., Angós I., Carrillo W. (2020). Analysis of environmental conditions effect in the phytochemical composition of potato (*Solanum tuberosum*) cultivars. Plants.

[38] Zhishen J., Mengcheng T., Jianming W. (1999). The determination of flavonoid contents in mulberry and their scavenging effects on superoxide radicals. Food Chem..

[39] Rapisarda P., Fanella F., Maccarone E. (2000). Reliability of analytical methods for determining anthocyanins in blood orange juices. J. Agric. Food Chem..

[40] Zambrano M., Vásquez G., Morales D., Vilcacundo R., Carrillo W. (2020). Isolation of baby lima bean (*Phaseolus lunatus* L.) proteins fractions and evaluation of their antioxidant activity. Ital. J. Food Sci..

[41] Babu D., Gurumurthy P., Borra S.K., Cherian K.M. (2013). Antioxidant and free radical scavenging activity of triphala determined by using different in vitro models. J. Med. Plants Res..

[42] Kalt W., McRae K.B., Hamilton L.C. (1995). Relationship between surface color and other maturity indices in wild lowbush blueberries. Can. J. Plant Sci..

[43] Tosun I., Ustun N.S., Tekguler B. (2008). Physical and chemical changes during ripening of blackberry fruits. Sci. Agric..

[44] Wills R.B.H., McGlasson W.B., Graham D., Joyce D.C. (1998). Structure and composition. Postharvest: An Introduction to the Physiology and Handling of Fruit, Vegetables and Ornamentals.

[45] Farinango M.E. (2010). Estudio de la Fisiología Postcosecha de la Mora de Castilla (*Rubus glaucus* Benth) y de la Mora Variedad Brazos (*Rubus* sp.). Bachelor’s Thesis.

[46] Brito B., Montalvo D., Freire V., Vásquez W., Viteri P., Martínez A., Jácome R., Galarza D., Garcés S., Velásquez J., Sánchez V., Zambrano J. (2016). Calidad en la cosecha, poscosecha y comercialización. El Cultivo de la Mora en el Ecuador.

[47] Vasco C., Ruales J., Kamal-Eldin A. (2008). Total phenolic compounds and antioxidant capacities of major fruits from Ecuador. Food Chem..

[48] Kaume L., Howard L.R., Devareddy L. (2012). The blackberry fruit: A review on its composition and chemistry, metabolism and biovailability, and health benefits. J. Agric. Food Chem..

[49] Mertz C., Gancel A.L., Gunata Z., Alter P., Dhuique-Mayer C., Vaillant F., Brat P. (2009). Phenolic compounds, carotenoids and antioxidant activity of three tropical fruits. J. Food Comp. Anal..

[50] Llerena W., Samaniego I., Ramos M., Brito B. (2014). Physico-chemistry and functional characterization of six tropical and Andean Ecuatorian fruits. Aliment. Cienc. Ing..

[51] Armijos D. (2018). Determinación del Contenido de Compuestos Bioactivos y Estudio de las Propiedades Antioxidantes en Extractos de mora Andina (*Rubus glaucus* Benth) de Ecuador. Bachelor’s Thesis.

[52] Ivanovic J., Tadic V., Dimitrijevic D., Stamenic M., Petrovic S., Zizovic I. (2014). Antioxidant properties of the anthocyanin-containing ultrasonic extract from blackberry cultivar “Cacanska Bestrna”. Ind. Crops Prod..

[53] Schulz M., Tischer S., Della Betta F., Nehring P., Camargo A., Daguer H., Valdemiro L., Oliveira A., Fett R. (2019). Blackberry (*Rubus Ulmifolius* Schott): Chemical composition, phenolic compounds and antioxidant capacity in two edible stages. Food Res. Int..

